# Radiological behavior and health risk assessment of radon gas in Lake Nasser sediments, Egypt: implications for natural hazards

**DOI:** 10.1007/s10653-025-02657-9

**Published:** 2025-08-03

**Authors:** Khaled Ali, Abd El-Baset Abbady, Ahmed Abu-Taleb, Shaban Harb

**Affiliations:** Physics Department, Faculty of Science, South Valley University, Qena, Egypt

**Keywords:** Natural hazards;, Radon;, Sediment analysis;, Radium content;, Exhalation rates;, Annual effective dose;, Radiological health risk;, and Health risk assessment.

## Abstract

Radon, a naturally occurring radioactive gas, represents a critical environmental factor linked to natural hazards through its contribution to radiation exposure in both indoor and outdoor environments. Therefore, it is essential to conduct an in-depth assessment of its dynamics in regions like Nasser Lake, Egypt. Radon activity concentrations were measured using the ionization chamber AlphaGUARD PQ2000PRO, while gamma spectrometry was employed to determine radium content in sediment samples. The results show significant variations in radon activity concentrations across the lake, ranging from 9.59 ± 0.37 Bq/m^3^ (lowest) to 66.24 ± 2.56 Bq/m^3^ (highest). A positive correlation was observed between radium content and radon activity concentrations. Radium content ranged from 10.37 ± 0.40 to 71.65 ± 2.77 Bq/kg. Radon exhalation rates for mass and area showed variability. The mass exhalation rates (× 10⁻^3^) ranged from 1.18 ± 0.05 to 8.13 ± 0.31 Bq/kg h. The area exhalation rates (× 10⁻^3^) ranged from 20.82 ± 0.81 to 143.84 ± 5.56 Bq/m^2^ h. The emanation coefficient (× 10⁻^3^) varied between 22.16 ± 0.86 and 214.98 ± 8.32, with an average value of 80.6 ± 3.12. These findings suggest a direct relationship between radium content and radon exhalation, emphasizing the role of sediment composition in radon release. The annual effective dose values, reflecting potential radiation exposure to residents and fishermen, ranged from 0.24 to 1.67 mSv/year, with an average of 0.57 mSv/year. These results fall mostly within the internationally accepted safety thresholds; however, certain locations near the High Dam showed elevated levels, indicating the need for continued monitoring in these areas. This study highlights the need for continuous environmental monitoring and risk mitigation strategies to reduce radon exposure. The findings enhance the understanding of radon dynamics in sediment-rich environments and support risk-informed decision-making frameworks related to environmental health and natural hazard mitigation.

## Introduction

Nasser Lake is one of the largest artificial reservoirs in the world and serves as a vital water resource for both Egypt and Sudan. In addition to its hydrological significance, it plays a vital role in electricity generation through the Aswan High Dam power station (Abd Ellah, 2020). The lake’s sedimentary environment has been shaped by decades of upstream sediment deposition, resulting in mineral-rich sediments that may contain elevated levels of naturally occurring radioactive materials (NORM) (Said, 2017). This presents potential environmental and health challenge, particularly in areas where local communities use these sediments for construction purposes (El-Shabrawy, 2014). Among the NORM present in the region, radon gas (^222^Rn) is of particular concern due to its radiological hazard potential (Din et al., 2020; Gümbür & Küçükönder, 2025; Küçükönder & Gümbür, 2022). ^222^Rn is a colorless, odorless, and tasteless noble gas produced from the decay of radium-226 (^226^Ra), which is commonly found in soils and rocks (Burkart, 1978; Din et al., 2020; Gümbür, 2024; Küçükönder et al., 2023). Long-term exposure to ^222^Rn and its decay products poses significant health risks, especially lung cancer, making it a major public health concern worldwide (National Research Council (US), 1988; Riudavets et al., 2022; Küçükönder & Gümbür, 2023). Although ^222^Rn occurs naturally in the environment, it can accumulate in enclosed spaces or in areas with high concentrations of radium-bearing minerals (Harb et al., 2016; International Atomic Energy Agency-IAEA, 2014). In the case of Nasser Lake, the sediments are rich in heavy minerals and clays, which tend to retain radium isotopes due to their adsorption properties (El-Taher & Abbady, 2012). These geochemical characteristics create favorable conditions for ^222^Rn accumulation and emanation. Additionally, fluctuations in water levels caused by seasonal changes and dam operations influence the redistribution of sediments and associated radionuclides, further affecting ^222^Rn dynamics (Ali et al., 2025; Darwish, 2013; Salahel Din et al., 2021). Local fishermen and residents often use these sediments to construct homes and fishing huts, particularly in areas affected by annual flooding. Rebuilding structures using such materials increases the risk of long-term ^222^Rn exposure, especially in poorly ventilated environments where the gas can accumulate (Szabo et al., 2012). ^222^Rn tends to concentrate in bottom sediments of lakes and aquatic systems due to natural sedimentation processes and the presence of radium-enriched particles (Beneš et al., 1986). Given the mineralogical composition of Nasser Lake's sediments, relatively high ^222^Rn levels are expected. Furthermore, climatic factors such as temperature and evaporation rates also affect ^222^Rn release and dispersion patterns (Imam et al., 2020). This study provides the first comprehensive assessment of ^222^Rn activity concentrations, exhalation rates, emanation coefficients, and annual effective doses in sediment samples collected from Nasser Lake, focusing on areas frequently used by local communities for construction. While previous studies have investigated natural radioactivity in Nile River sediments and coastal regions of Egypt (Beneš et al., 1986; Darwish, 2013; El-Taher & Abbady, 2012; Imam et al., 2020; Szabo et al., 2012), this research uniquely combines spatial analysis, radiological measurements, and health risk assessment tailored to the specific geological and social context of Nasser Lake. By doing so, it contributes to the understanding of ^222^Rn behavior in sediment-rich environments and supports evidence-based decision-making for environmental and public health protection.

## Experimental settings

### Sampling

Thirty shore sediment samples were collected around Nasser Lake at the end of the spring season, using a grab sampler from a distance of 5–10 meter (m) from the shore, at a water depth ranging from 0.5 to 1 m. These sites were selected based on the spatial distribution in areas where residents and fishermen are intensively engaged in activities such as using sediments for constructing houses and huts near the shore. The locations were also selected based on environmental characteristics that may contribute to the accumulation of radioactive elements, such as radium, in these regions. After collecting, the samples were transferred to the laboratory in airtight plastic bags to maintain their original composition and prevent contamination. Stones and other unwanted materials were removed from the samples to ensure that only fine sediments were analyzed. Subsequently, the samples were air-dried for 2–3 days in stainless-steel trays to begin removing surface moisture (Knoll, 2010). Afterward, they were completely dried in an oven at a constant temperature of 105 °C for 24 hours, a temperature that ensures complete moisture removal without affecting the mineral composition of the samples. This step is essential to prevent any interference caused by the presence of water during ^222^Rn measurements (Knoll, 2010). Once dried, the samples were ground using an electric mill to achieve a fine, homogeneous powder. Grinding to a fine powder increases the surface area and improves the accuracy of ^222^Rn measurements. Each sample was then mixed thoroughly using an electric shaker to ensure homogeneity. The powder was shaped into a cone and then divided into four quarters using the quartering method, a well-established academic procedure for evenly dividing samples (Conklin & Alfred, 2004). Opposite quarters were combined and mixed thoroughly, and the process was repeated until the desired weight of the sample was obtained, ensuring that it accurately represented the original sample. To allow for radioactive equilibrium between ^226^Ra and ^222^Rn, the prepared samples were sealed in airtight containers and stored for a minimum period of four weeks before measurement (Knoll, 2010). This method ensures that the samples are homogeneous and representative, which is crucial for obtaining reliable results when performing ^222^Rn measurements using the ionization chamber AlphaGUARD PQ2000PRO detector (Harb et al., 2016; Salaheldin et al., 2022).

### Measurement technique

**Fig. 1:**
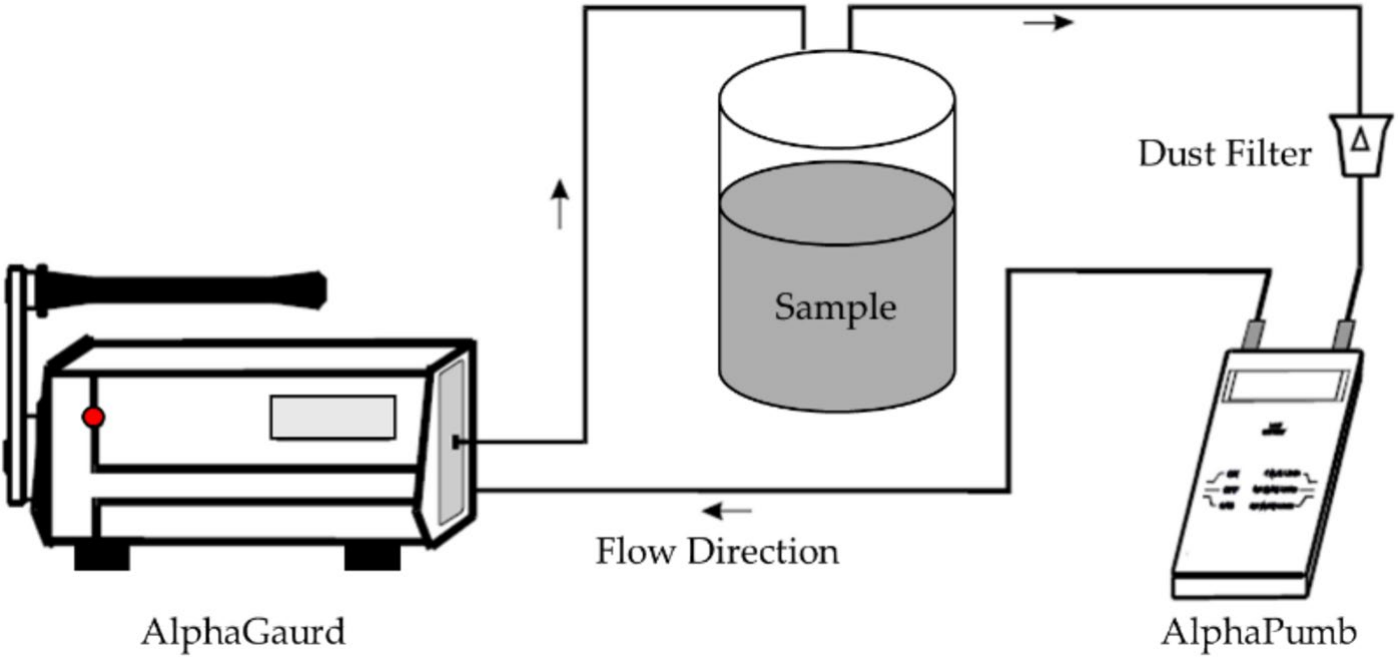
Application of AlphaPUMP in a closed gas cycle (emanation measurement in a closed vessel). (Ali et al. 2025)

The radioactivity of natural isotopes, ^226^Ra, thorium-232 (^232^Th), and potassium-40 (^40^K), in shore sediment samples from Nasser Lake was measured using a sodium iodide NaI(Tl) gamma spectrometer with a thallium-doped scintillation counter. These measurements were published in a previous study for our group (Taleb et al., 2019). Since ^226^Ra is the parent isotope that leads to the emission of ^222^Rn, the results of the radioactivity measurements of ^226^Ra will be utilized in the current study. This is crucial for determining ^222^Rn concentrations and their environmental and health impacts in the region. In this study, the AlphaGUARD detector, along with the special AquaKIT equipment system, was used to measure the exhalation rates of ^222^Rn from shore sediment samples (AS-Subaihi & Salem, 2020; Harb et al., 2016; Salaheldin et al., 2022). The system consists of degassing vessels, an AlphaPUMP, an AlphaGUARD, and the progeny filter. Although the AquaKIT system is primarily designed for measuring ^222^Rn in water samples (Din et al., 2020), it was modified and adapted in this study to measure ^222^Rn exhalation from solid sediment samples using a closed-gas-circulation setup. In this configuration, the sediment sample was placed inside a sealed degassing vessel, and the air within the system was continuously circulated using the AlphaPUMP. This allowed ^222^Rn to be released from the solid matrix into the gas phase, where it was then detected by the AlphaGUARD detector. The principle of measurement relies on the exhalation of ^222^Rn from the sediment under controlled conditions, enabling accurate quantification of ^222^Rn exhalation rates. Before starting the measurements, the system is flushed with normal air until the ^222^Rn values are comparable to ambient air concentrations (Din et al., 2020). The background of the empty system is measured for a few minutes before each sample measurement. After that, the sample is introduced into the degassing vessel, and the AlphaPUMP is switched on to pump the air continuously, separating ^222^Rn from the sample. After 10 min, the AlphaPUMP is turned off, and the AlphaGUARD continues measuring for an additional 20 min. The measurements are conducted in a flow mode, with the ^222^Rn concentration recorded every 10 min. The AlphaPUMP was set to a flow rate of 0.3 L/min throughout the measurement process. This cycle was repeated three times to ensure better precision in the results. The AlphaGUARD PQ2000PRO detector operates within a wide dynamic measurement range of 0–100,000 Bq/m^3^ for ^222^Rn activity concentration, making it suitable for both low- and high-level environmental measurements. The detector was calibrated by the manufacturer (Genitron Instruments GmbH) under controlled laboratory conditions, traceable to national standards. Field calibration checks were also performed before each measurement session using an empty vessel to establish background levels, ensuring high accuracy and repeatability throughout the experimental campaign. This focus on ^222^Rn measurements is particularly important, as the results will be used as a reference for the ^222^Rn-related radioactivity in the environmental samples. Figure 1 illustrates the measurement system used in the experiments conducted with the AlphaGUARD detector (Harb et al., 2016).

### Analytical methodology

Before proceeding with the calculation and analysis of the various parameters measured in this study, it is necessary to outline the scientific approach employed to assess the ^222^Rn concentrations, radium content (Co_*Ra*_), and associated radioactivity in the collected sediment samples. This study aims to provide precise and reliable data on the radiological properties of the shore sediments from Nasser Lake, using advanced instruments such as the AlphaGUARD detector and gamma spectrometer for accurate measurement of radioactive activity. The measurements were conducted considering environmental factors such as temperature and humidity, which can affect the precision of ^222^Rn and ^226^Ra detection. Representative sediments were obtained to ensure a thorough and spatially relevant analysis of ^222^Rn emanation and Co_*Ra*_. The collected samples were then analyzed for radioactivity using the appropriate techniques, considering the necessary calibration, and ensuring that all measurements were dependable. Based on the methodology outlined below, calculations were performed to determine key parameters such as mass exhalation rate (ExM), area exhalation rate (ExA), emanation factor (EmC), and annual effective dose (AED). These parameters provide valuable insights into the behavior of ^222^Rn and its progeny in sediment and their potential impact on both the environment and human health (Riudavets et al., 2022). The approach used to determine each parameter follows established methodologies in the field of environmental radiology, ensuring the accuracy and consistency of the results. In the following sections, detailed descriptions of the calculation methods for each parameter, along with the respective instruments and data analysis procedures, are provided. The methodology outlined in this study ensures that the data obtained is of high quality and accurately represents the radiological conditions of the study area, contributing to the understanding of ^222^Rn behavior in sediment samples from Lake Nasser.

#### ^222^Rn activity concentration

The ^222^Rn concentration at time (t), denoted as Rn(t), was continuously monitored within the AlphaGUARD detector. During the measurement process, Rn(t) is related to its equilibrium concentration (Rn_*eq*_) as the ^222^Rn released from each sample accumulates in the closed chamber. Rn_*eq*_ represents the stable ^222^Rn concentration reached after a sufficient period of time, typically after 30 days, allowing for the saturation of the sealed space. Rn_*eq*_ for each sample was calculated using the following exponential growth relation of Eq. (1) (Harb et al., 2016; Salaheldin et al., 2022):

1 $$Rn\left( t \right) = Rn_{eq} \times \left( {1 - e^{ - \lambda t} } \right)$$

where $Rn\left( t \right)$ and $Rn_{eq}$ are expressed in Bq/m^3^, t is the accumulation time of the ^222^Rn released from the sample, measured in seconds, λ is the decay constant of ^222^Rn, and t ≫ 30 days for equilibrium conditions to be fully established. This equation accounts for the time-dependent growth of ^222^Rn within the chamber until it reaches a constant value corresponding to the equilibrium concentration. The $Rn_{eq}$ is a key radiological parameter for assessing the potential radiation exposure over extended periods. The equation is based on the principle that the ^222^Rn concentration increases over time, approaching the equilibrium value as the sample continuously releases ^222^Rn (Poinssot & Geckeis, 2015).

#### Radium content

Co_*Ra*_ of a sample is an important factor influencing environmental ^222^Rn levels, as ^222^Rn is produced from the decay of ^226^Ra through alpha-decay. Co_*Ra*_ determined by measuring the ^222^Rn concentration emitted from the sample inside the measurement chamber of AlphaGUARD detector. To calculate the effective Co_*Ra*_ of the sample, expressed in Bq/kg, the following Eq. (2) was used (Harb et al., 2016; Salaheldin et al., 2022):

2 $$Co_{Ra} = \frac{Rn\left( t \right) \times V}{M}$$

where M is the mass of the solid sample in kg, and V is the volume of the cylindrical vessel used for the measurement in m^3^. Through this equation, the relationship between the ^222^Rn concentration emitted from the sample and the Co_*Ra*_ is established, allowing for the assessment of the radiological activity generated by this element and estimating the potential ^222^Rn levels in the surrounding environment (International Atomic Energy Agency-IAEA, 2014).

#### ^222^Rn exhalation rates

ExM and ExA were calculated to assess the rate at which ^222^Rn is emitted from the sample. These measurements provide important insights into the ^222^Rn release from various materials, which is essential for understanding environmental ^222^Rn levels. ExM, which indicates the amount of ^222^Rn released per unit mass of the sample, is calculated using Eq. (3) as follows (International Commission on Radiological Protection-ICRP, 1993):

3 $${\text{E}\times \text{M}}= { }\frac{{{\text{Rn}}_{{{\text{eq}}}} { } \times {\uplambda } \times {\text{V }}}}{{\text{M}}}$$

This equation quantifies the rate of ^222^Rn release in relation to the mass of the material, providing an indicator of how the material contributes to ^222^Rn exposure based on its mass. E × A, which measures the amount of ^222^Rn emitted per unit surface area of the sample, is given by Eq. (4) as follows (Harb et al., 2016; International Commission on Radiological Protection-ICRP, 1993):

4 $${\text{E}\times\text{A}}= { }\frac{{{\text{Rn}}_{{{\text{eq}}}} { } \times {\uplambda } \times {\text{V }}}}{{\text{A}}}$$

where A is the surface area of the sample in m^2^, which is equivalent to the cross-sectional area of the emanation container. This rate provides an estimate of the ^222^Rn release based on the surface area of the sample, offering an important measure of the sample’s potential to release ^222^Rn into the surrounding environment (AS-Subaihi & Salem, 2020; Yang et al., 2019).

#### Emanation coefficient

EmC is a measure of a material's efficiency in releasing ^222^Rn as a result of the decay of ^226^Ra within the sample. This coefficient is determined based on the relationship between the volume of the chamber used to measure the emissions and the ^226^Ra activity concentration (A_*Ra*_) present in the sample. EmC is an important tool for understanding the behavior of radioactive emissions from various samples and assessing their environmental impact. It is primarily used in environmental research to determine ^222^Rn exhalation levels from different materials such as soil, rock, or building materials, helping evaluate the potential effects of naturally occurring radioactive materials on the surrounding environment and human health. EmC is calculated using Eq. (5) as follows (International Commission on Radiological Protection-ICRP, 1993; Salaheldin, 2019):

5 $${\text{EmC}} = \frac{{{\text{V}} \times {\text{Rn}}_{{{\text{eq}}}} }}{{{\text{M}} \times {\text{A}}_{{{\text{Ra}}}} }}$$

This equation is a key tool for determining a material's ability to release ^222^Rn based on its physical and radiological properties (Raslan et al., 2010).

#### Annual effective dose

AED for local inhabitants was calculated using the experimentally determined Rn_eq_. These values were obtained by measuring ^222^Rn accumulation in a sealed chamber over time until equilibrium conditions were reached (~ 30 days), ensuring that the Rn_eq_ values reflect actual ^222^Rn exhalation from the sediment samples under controlled conditions. The calculation follows the methodology provided by the United Nations Scientific Committee on the Effects of Atomic Radiation (UNSCEAR), as shown in Eq. (6) (United Nations Scientific Committee on the Effects of Atomic Radiation (UNSCEAR), 2000):

6 $${\text{AED }} = {\text{Rn}}_{{{\text{eq}}}} \times {\text{Ef}} \times {\text{Of}} \times {\text{DCf}}$$

where Rn_*eq*_ is the ^222^Rn concentration at equilibrium, measured in Bq/m^3^, Ef is the equilibrium factor between ^222^Rn and its short-lived decay products, taken as 0.4 according to UNSCEAR recommendations (United Nations Scientific Committee on the Effects of Atomic Radiation (UNSCEAR), 2000), Of is the indoor occupancy factor, representing the average time individuals spend indoors per year (7000 h/year), and DCf is the dose conversion factor, set at 9.0 nSv/h per Bq/m^3^, which quantifies the radiation dose from ^222^Rn and its progeny. This equation estimates the radiation dose received by individuals due to inhalation exposure to ^222^Rn and its progeny under typical indoor conditions (Abbady et al., 2025; Abbas et al., 2020; Abumurad, 2024; Baldık et al., 2011; Din et al., 2020; Hassan et al., 2011; International Commission on Radiological Protection, 1987; Özden & Pehlivanoğlu, 2025; Öztürk, 2022; Pervin et al., 2022; Salaheldin et al., 2022; Sola et al., 2015; Thabayneh & Gharaybeh, 2023). It accounts for the ^222^Rn concentration, internal equilibrium factors, and annual exposure duration, providing a realistic and accurate estimation of the radiation risk faced by residents and fishermen who are frequently exposed to sediments with elevated ^222^Rn levels, particularly in confined spaces such as homes or fishing huts constructed from these materials.

## Results and discussion

This section delves into the radiological parameters obtained from the sediment of Nasser Lake, focusing on Rn_*eq*_, Co_*Ra*_, ExM, ExA, EmC, and the AED. These parameters were meticulously selected due to their critical importance in understanding the radiological behavior of ^222^Rn and its progeny within environmental contexts. ^222^Rn is a well-known contributor to indoor and outdoor radiation exposure. Assessing its concentration and behavior provides valuable insights into potential radiological risks to human health, particularly for residents and fishermen who are frequently exposed to these sediments. In regions where sediments are used by these populations to construct small houses and fishing huts, the potential for exposure to ^222^Rn is a concern that must be addressed. The parameters analyzed in this study are interconnected, offering a comprehensive evaluation of ^222^Rn generation, release, and potential exposure pathways. For instance, Co_*Ra*_ serves as the primary source of ^222^Rn within the sediments, while ExM and ExA quantify how efficiently ^222^Rn escapes from the material into the atmosphere. The ExM and ExA measurements are especially relevant to understanding how much ^222^Rn these local communities may be exposed to in the areas where they live and work. The EmC provides insights into the fraction of ^222^Rn atoms released relative to the ^226^Ra present, reflecting the physical and chemical characteristics of the sediments that are being used in construction activities. Finally, the AED translates these radiological properties into a tangible measure of potential exposure risks for individuals living near the study area, especially those involved in construction and fishing activities. The selection of these parameters stems from their role in bridging the gap between raw radiological data and its environmental and health implications. By analyzing these factors in tandem, this study provides a holistic understanding of ^222^Rn dynamics in the sedimentary environment of Lake Nasser, with a particular focus on the potential risks to the local communities and their health. This contributes to the broader discourse on radiological safety and environmental monitoring, highlighting the need for effective risk mitigation strategies. In the following sections, each parameter will be discussed in detail, emphasizing its significance, trends observed in the results, and the implications for environmental and public health. The results of those parameters are shown in Table 1.

**Table 1:** Activity concentrations of ^226^Ra, Rn_eq_, radium contents, ^222^Rn exhalation rates (mass and area), emanation coefficients, and the annual effective dose

No.	Coordinates	^226^Ra (Bq/kg)	Rn_eq_ (Bq/m^3^)	Co_Ra_ (Bq/kg)	EXM × 10^–3^ (Bq/k.h)	EXA × 10^–3^ (Bq/m^2^.h)	EmC × 10^–3^	AED (mSv/year)
S1	23° 58′ 11″ N 32° 55′ 18″ E	4.15 ± 0.16	16.39 ± 0.63	17.62 ± 0.68	2.00 ± 0.08	35.37 ± 1.37	63.79 ± 2.47	0.41
S2	23° 53′ 25″ N 32° 43′ 35″ E	4.25 ± 0.16	38.49 ± 1.49	41.51 ± 1.61	4.71 ± 0.18	83.32 ± 3.22	146.64 ± 5.67	0.97
S3	23° 46′ 53″ N 32° 49′ 49″ E	5.00 ± 0.20	66.24 ± 2.56	71.65 ± 2.77	8.13 ± 0.31	143.84 ± 5.56	214.98 ± 8.32	1.67
S4	23° 51′ 45″ N 32° 47′ 53″ E	11.00 ± 0.42	64.94 ± 2.51	70.36 ± 2.72	7.98 ± 0.31	141.24 ± 5.46	96.01 ± 3.71	1.64
S5	23° 55′ 35″ N 32° 55′ 26″ E	17.55 ± 0.68	35.11 ± 1.36	38.07 ± 1.47	4.32 ± 0.17	76.43 ± 2.96	32.56 ± 1.26	0.88
S6	23° 08′ 37″ N 32° 51′ 34″ E	7.58 ± 0.29	25.16 ± 0.97	27.20 ± 1.05	3.09 ± 0.12	54.60 ± 2.11	53.87 ± 2.08	0.63
S7	23° 08′ 37″ N 32° 51′ 34″ E	4.83 ± 0.19	36.99 ± 1.43	39.89 ± 1.54	4.53 ± 0.18	80.08 ± 3.10	123.83 ± 4.79	0.93
S8	23° 54′ 48″ N 32° 46′ 41″ E	6.89 ± 0.27	22.32 ± 0.86	24.00 ± 0.93	2.72 ± 0.11	48.17 ± 1.86	52.25 ± 2.02	0.56
S9	23° 40′ 08″ N 32° 31′ 36″ E	4.46 ± 0.17	23.25 ± 0.9	25.07 ± 0.97	2.84 ± 0.11	50.33 ± 1.95	84.45 ± 3.27	0.59
S10	23° 08′ 55″ N 32° 38′ 59″ E	7.03 ± 0.27	9.59 ± 0.37	10.37 ± 0.40	1.18 ± 0.05	20.82 ± 0.81	22.16 ± 0.86	0.24
S11	23° 17′ 53″ N 32° 44′ 04″ E	5.04 ± 0.20	32.36 ± 1.25	35.06 ± 1.36	3.98 ± 0.15	70.38 ± 2.72	104.33 ± 4.04	0.82
S12	23° 29′ 29″ N 33° 01′ 43″ E	6.15 ± 0.24	19.18 ± 0.74	20.80 ± 0.80	2.36 ± 0.09	41.75 ± 1.62	50.79 ± 1.96	0.48
S13	23° 53′ 58″ N 32° 54′ 36″ E	4.29 ± 0.17	18.13 ± 0.70	19.60 ± 0.76	2.22 ± 0.09	39.35 ± 1.52	68.62 ± 2.65	0.46
S14	23° 49′ 51″ N 32° 56′ 16″ E	2.62 ± 0.10	19.42 ± 0.75	20.94 ± 0.81	2.38 ± 0.09	42.04 ± 1.63	120.09 ± 4.65	0.49
S15	22° 31′ 40″ N 31° 54′ 23″ E	2.47 ± 0.10	18.38 ± 0.71	19.76 ± 0.76	2.24 ± 0.09	39.67 ± 1.53	120.06 ± 4.64	0.46
S16	23° 02′ 16″ N 32° 35′ 18″ E	2.50 ± 0.10	10.16 ± 0.39	10.96 ± 0.42	1.24 ± 0.05	21.99 ± 0.85	65.89 ± 2.55	0.26
S17	22° 35′ 19″ N 31° 48′ 32″ E	2.46 ± 0.10	11.84 ± 0.46	12.81 ± 0.50	1.45 ± 0.06	25.71 ± 0.99	78.23 ± 3.03	0.30
S18	22° 37′ 34″ N 32° 21′ 55″ E	4.34 ± 0.17	15.55 ± 0.60	16.85 ± 0.65	1.91 ± 0.07	33.82 ± 1.31	58.31 ± 2.26	0.39
S19	22° 27′ 45″ N 31° 40′ 32″ E	4.43 ± 0.18	15.92 ± 0.62	17.26 ± 0.67	1.96 ± 0.08	34.66 ± 1.34	58.52 ± 2.26	0.40
S20	22° 24′ 11″ N 31° 52′ 22″ E	4.82 ± 0.19	13.98 ± 0.54	15.11 ± 0.58	1.71 ± 0.07	30.34 ± 1.17	47.02 ± 1.82	0.35
S21	22° 34′ 52″ N 31° 39′ 54″ E	1.93 ± 0.08	16.39 ± 0.63	17.67 ± 0.68	2.01 ± 0.08	35.48 ± 1.37	137.55 ± 5.32	0.41
S22	22° 15′ 33″ N 31° 29′ 40″ E	3.60 ± 0.14	25.29 ± 0.98	27.19 ± 1.05	3.08 ± 0.12	54.58 ± 2.11	113.36 ± 4.38	0.64
S23	22° 45′ 53″ N 32° 31′ 44″ E	4.10 ± 0.16	13.82 ± 0.53	14.90 ± 0.58	1.69 ± 0.07	29.92 ± 1.16	54.60 ± 2.11	0.35
S24	22° 27′ 53″ N 32° 01′ 39″ E	4.58 ± 0.18	13.03 ± 0.50	14.09 ± 0.55	1.60 ± 0.06	28.29 ± 1.09	46.14 ± 1.78	0.33
S25	23° 02′ 16″ N 32° 35′ 18″ E	4.14 ± 0.17	21.92 ± 0.85	23.75 ± 0.92	2.69 ± 0.10	47.67 ± 1.84	86.00 ± 3.33	0.55
S26	22° 51′ 04″ N 32° 10′ 57″ E	4.45 ± 0.15	10.59 ± 0.41	11.48 ± 0.44	1.3 ± 0.05	23.05 ± 0.89	38.69 ± 1.50	0.27
S27	22° 15′ 14″ N 31° 35′ 57″ E	2.44 ± 0.10	13.27 ± 0.51	14.35 ± 0.55	1.63 ± 0.06	28.80 ± 1.11	88.27 ± 3.41	0.33
S28	22° 47′ 44″ N 32° 14′ 28″ E	5.45 ± 0.21	23.94 ± 0.93	25.82 ± 1.00	2.93 ± 0.11	51.83 ± 2.00	71.09 ± 2.75	0.60
S29	22° 21′ 46″ N 31° 47′ 12″ E	4.29 ± 0.17	13.48 ± 0.52	14.49 ± 0.56	1.64 ± 0.06	29.09 ± 1.13	50.73 ± 1.96	0.34
S30	22° 29′ 08″ N 31° 47′ 00″ E	3.97 ± 0.16	17.01 ± 0.66	18.34 ± 0.71	2.08 ± 0.08	36.82 ± 1.42	69.33 ± 2.68	0.43
Minimum		1.93 ± 0.08	9.59 ± 0.37	10.37 ± 0.40	1.18 ± 0.05	20.82 ± 0.81	22.16 ± 0.86	0.24
Maximum		17.55 ± 0.68	66.24 ± 2.56	71.65 ± 2.77	8.13 ± 0.31	143.84 ± 5.56	214.98 ± 8.32	1.67
Mean		5.03 ± 0.2	22.74 ± 0.88	24.57 ± 0.95	2.79 ± 0.11	49.32 ± 1.91	80.6 ± 3.12	0.57

### Discussion of ^222^Rn concentrations in the sediments of Nasser Lake

**Fig. 2:**
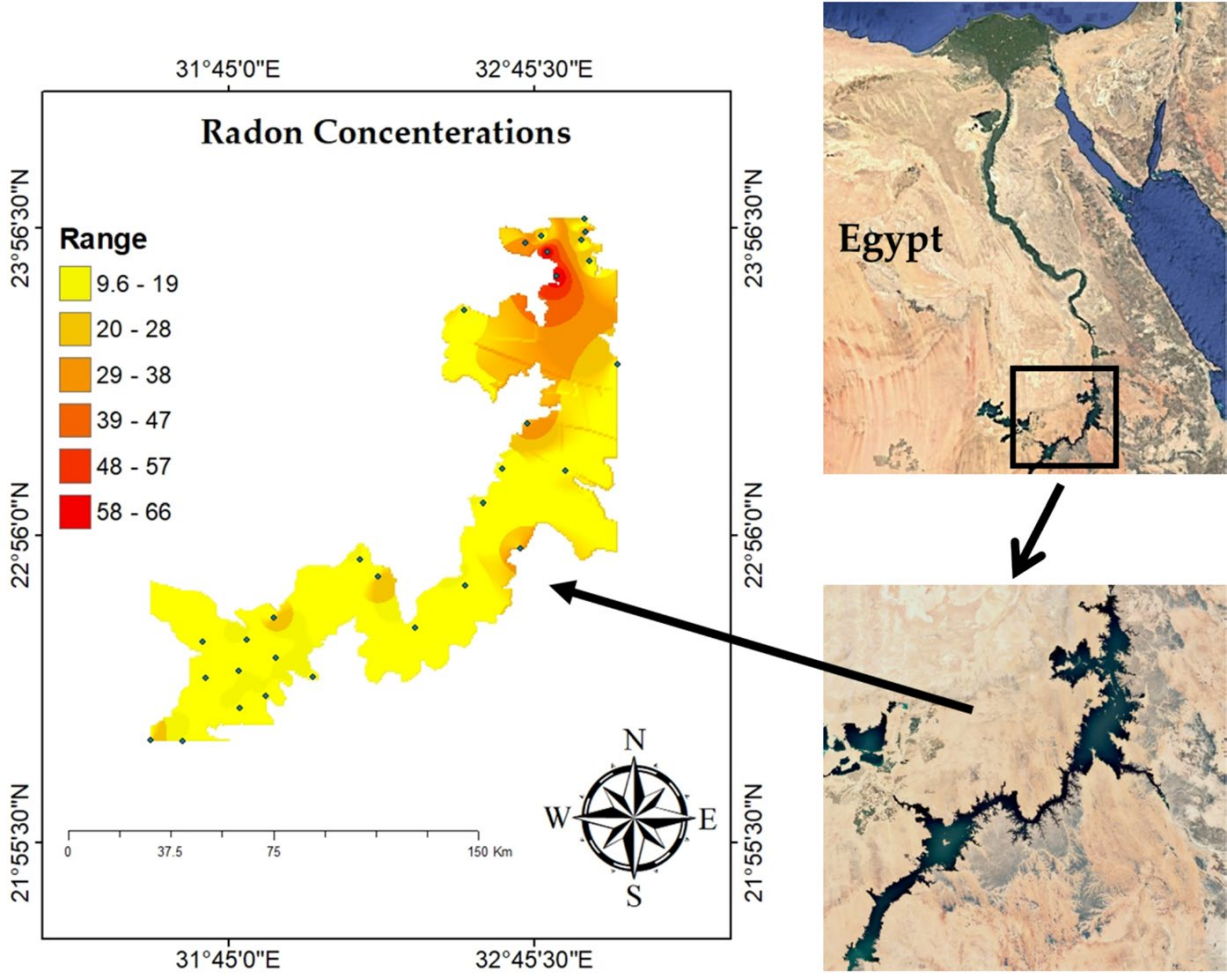
Spatial distribution of ^222^Rn concentrations (Bq/m^3^) in Lake Nasser sediments using geographic information systems (GIS)

**Fig. 3:**
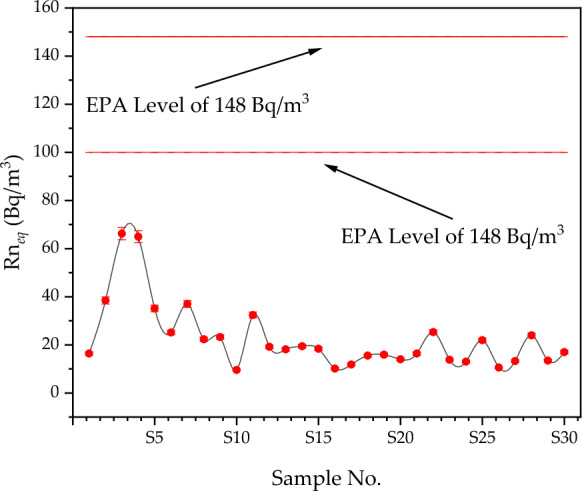
^222^Rn concentrations in different sampling locations across Lake Nasser, Egypt

^222^Rn concentrations in the sediment of Nasser Lake show significant variation across different sampling locations, likely influenced by various geological and environmental factors. These factors include sediment composition and the presence of radium-rich materials, which are known to contribute to ^222^Rn accumulation and emanation. A spatial analysis was conducted using geographic information systems (GIS) to better understand the distribution of ^222^Rn concentrations across the study area. The results of this analysis indicated that certain regions of Nasser Lake exhibit higher ^222^Rn levels, particularly near areas where sediment deposition occurs closer to the High Dam. This suggests a direct correlation between sediment accumulation and ^222^Rn concentration. Sediments in the lower part of the lake, before the dam, tend to be more concentrated with radioactive materials like ^226^Ra, which decays to release ^222^Rn. The High Dam acts as a barrier preventing sediment flow, thereby contributing to the accumulation of these radioactive materials in this area. Additionally, the region may have experienced a higher accumulation of radioactive materials due to its specific geological characteristics, such as rocks or soil rich in radioactive elements that lead to ^222^Rn accumulation. Fluctuations in water levels, influenced by the dam, also contribute to the accumulation of radioactive materials in this area, resulting in increased ^222^Rn concentrations. GIS mapping, as shown in Fig. 2, provides a clear visual representation of how ^222^Rn levels distribute spatially across the lake. Figure 3 reveals the highest recorded concentration of 66.24 ± 2.56 Bq/m^3^ at sample S3 (23° 46′ 53″ N 32° 49′ 49″ E), while the lowest concentration of 9.59 ± 0.37 Bq/m^3^ was found at sample S10 (23° 08′ 55″ N 32° 38′ 59″ E). These findings suggest that areas closer to the High Dam, where sediment accumulation is prominent, have higher ^222^Rn levels, further supporting the correlation between sediment deposition and ^222^Rn levels. Local populations and fishermen, who use the sediments for the construction of houses and other structures, may experience prolonged exposure to ^222^Rn. While most measured annual effective doses fall within internationally accepted safety thresholds, certain locations near the High Dam showed slightly elevated values, highlighting the importance of continuous monitoring to ensure public health safety and environmental protection. While the levels of ^222^Rn in the lake fall within internationally accepted safety limits, with values below the World Health Organization (WHO) threshold of 100 Bq/m^3^ (World Health Organization, 2009) and the U.S. Environmental Protection Agency (EPA) action level of 148 Bq/m^3^ (U.S. Environmental Protection Agency, n.d.), the continuous exposure to local populations necessitates periodic monitoring to ensure safety. In conclusion, the spatial analysis of ^222^Rn concentrations in Nasser Lake sediments highlights the relationship between sediment accumulation and ^222^Rn levels, with higher concentrations found near the High Dam where sediment deposition occurs. The geological characteristics of this area, combined with the influence of the High Dam, contribute to the accumulation of ^222^Rn-rich sediments. The potential long-term exposure risks to local populations emphasize the importance of continuous monitoring to ensure both environmental and public health safety.

A comparison with previously published studies on ^222^Rn concentrations in different geological and construction materials was conducted to provide a broader context for our findings. As shown in Table 2, the Rn_*eq*_ in this study ranges from 9.59 ± 0.37 to 66.24 ± 2.56 Bq/m^3^, which is significantly lower than values reported for granitic rocks from other Egyptian regions (Salaheldin et al., 2022), where concentrations reached up to 251.40 Bq/m^3^. The measured Rn_*eq*_ levels are also below those recorded for industrial by-products such as phosphor gypsum used in building applications (Maged & Ashraf, 2005), but fall within the range observed for common construction materials like gypsum, ceramic, marble, and sand (Gupta et al., 2009; Yousef et al., 2015). These comparisons suggest that Lake Nasser sediments generally exhibit low ^222^Rn emission potential compared to other natural and man-made sources. However, certain locations near the High Dam showed relatively higher values, indicating the need for radiological screening when using these sediments in residential construction. This comparative analysis provides a useful reference for understanding regional variations in ^222^Rn behavior and supports evidence-based risk assessment in sediment-rich environments.

**Table 2:** Comparison of ^222^Rn concentrations in Lake Nasser sediments and other environmental or building materials globally

Country	Region (material type)	^222^Rn (Bq/m^3^)	Reference study
Egypt	Nasser Lake (shore sediments)	9.59 ± 0.37 to 66.24 ± 2.56	This study
	El-Missikat (granitic rocks)	49.60–107.94	Salaheldin et al. (2022)
	Kab-Amira (granitic rocks)	33.21–251.40	
	Cairo (building materials)	24–55	Maged and Ashraf (2005)
	Northeastern Desert (soil and rock)	2470 to 19.200	Salaheldin (2019)
	Sand	Avg. (24.00)	El-Arabi et al. (2006)
	Granite	up to 250	
	Black cement	Avg. (200.47)	Yousef et al. (2015)
	White cement	Avg. (68.42)	
	Gypsum	Avg. (80.67)	
	Marble	Avg. (219.18)	
	Bricks	Avg. (127.06)	
	Stone	Avg. (320.83)	
	Gravel	Avg. (116.47)	
	Ceramic	Avg. (95.29)	
Turkey	Indoor building materials	66 to 1711	Öztürk (2022)
	Soil samples	62.87 to 421.897	Küçükönder et al. (2023)
Libya	Brick walls	7.8 to 277.9, Avg. (72 ± 5.8)	Saad et al. (2010)
	Marble ledges	100.0 to 298.7, Avg. (174.5 ± 5.8)	
	Ceramic floors	7.0 to 275.3, Avg. (avg: 145.1 ± 4.9)	
India	Stone	Avg. (731)	Gupta et al. (2009)
	Cement	Avg. (474)	
Global	Global background	~ 10–30	United Nations Scientific Committee on the Effects of Atomic Radiation (UNSCEAR) (2000)
	Indoor exposure limit	200–600	International Commission on Radiological Protection-ICRP (1993)

### Radium content and its correlation with ^222^Rn concentration in the sediments of Nasser Lake

**Fig. 4:**
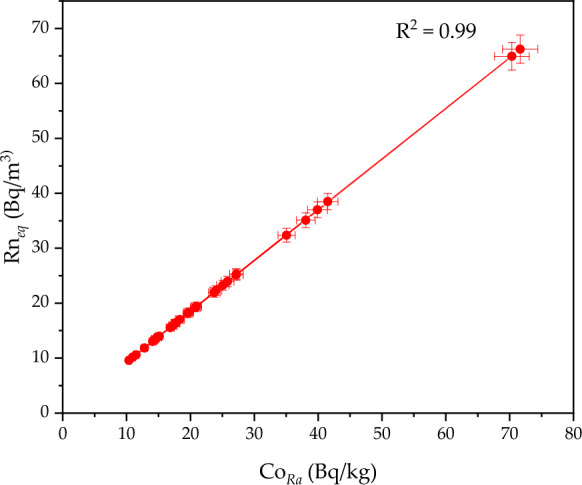
Radium content versus ^222^Rn concentration in the sediments of Nasser Lake

Co_*Ra*_ in sediments is a key factor influencing environmental ^222^Rn levels, as ^222^Rn is produced from the decay of ^226^Ra. Therefore, measuring Co_*Ra*_ in sediments is essential for understanding ^222^Rn emissions. The Co_*Ra*_ values in Nasser Lake sediments showed variability, ranging from 10.37 ± 0.40 to 71.65 ± 2.77 Bq/kg, with an average of 24.57 ± 0.95 Bq/kg. This variability reflects the geographical and geological differences that may influence the accumulation of ^226^Ra in sediments, especially in regions close to the shore where local fishermen and residents tend to gather sediments for building small homes and huts. These regions, typically impacted by the annual fluctuations in the lake's water levels due to seasonal water level variations, contribute to the accumulation and use of radium-rich materials in construction. The analysis of the relationship between Co_*Ra*_ and Rn_*eq*_, illustrated in Fig. 4, demonstrated a strong positive correlation. An increase in Co_*Ra*_ corresponded to a rise in Rn_*eq*_ within the samples, confirming that ^222^Rn originates from the decay of ^226^Ra in sediments. The plot further highlighted this linear correlation, with a near-perfect relationship approaching + 1. Additionally, no outliers were observed in the plot, which reinforces the accuracy of the analysis and the reliability of the relationship between the parameters. These findings are significant for understanding the distribution of ^222^Rn in the local environment, particularly in areas where human activity, such as the construction of homes and huts by local communities, may lead to moderate increases in ^222^Rn exposure levels. By determining the Co_*Ra*_, it is possible to estimate potential ^222^Rn levels, particularly in regions with high sediment accumulation used for building purposes. This study also contributes to improving strategies for monitoring and controlling radiation concentrations in the surrounding environment, especially in areas where the local population is exposed to higher risks due to their interaction with the sediments. The reference values for Co_*Ra*_ in sediments vary depending on the geographical region and local environmental standards. In the case of sediment samples from Nasser Lake, the accumulation of radium in the sediments reflects the impact of surrounding rocks and geological formations, as well as human activity in the area, such as the use of these sediments for construction purposes. Although no specific standards for Co_*Ra*_ in sediments are set by environmental protection agencies, elevated levels of ^226^Ra in sediments can be indirectly linked to ^222^Rn accumulation in the surrounding area, especially in regions where such sediments are regularly disturbed by human activities.

### ^222^Rn exhalation rates: Dynamics and environmental implication

**Fig. 5:**
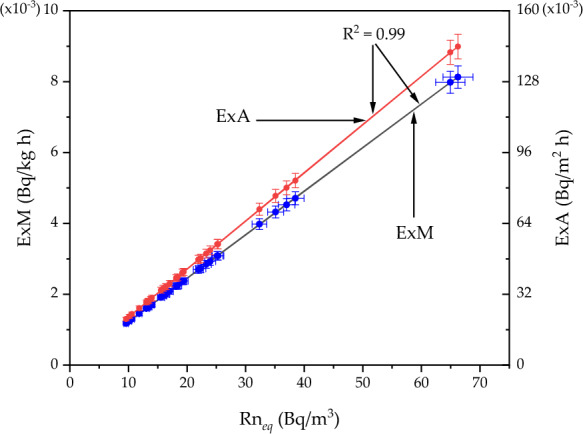
^222^Rn concentration versus the exhalation rates (mass and area) in the sediments of Nasser Lake

Continuing the analysis of ^222^Rn behavior in the sediments of Nasser Lake, examining the exhalation rates (ExM and ExA) represents a logical extension of the previous results on Rn_*eq*_ and Co_*Ra*_. These parameters provide significant insights into the dynamics of ^222^Rn release into the environment, which is particularly important in regions with high geological and environmental variability, such as the study area. The study area, where residents and fishermen frequently use sediments for constructing small homes and fishing huts, particularly in areas exposed to seasonal fluctuations in water levels, makes this investigation even more relevant. Understanding ^222^Rn exhalation rates is essential for assessing potential exposure and the associated radiological risks, particularly in regions where sediment is routinely used in construction activities. The ExM (× 10^⁻3^) ranged between 1.18 ± 0.05 and 8.13 ± 0.31 Bq/kg·h, reflecting variability in the samples'capacity to release ^222^Rn. This variability is influenced by their internal characteristics and Co_*Ra*_, with some areas near settlements or construction zones likely exhibiting higher exhalation rates due to the direct use of the sediments. The average ExM rate was 2.79 ± 0.11 Bq/kg·h, which serves as a baseline for evaluating other samples from the same geological context, particularly in areas near inhabited zones. The ExA (× 10^⁻3^) exhibited a broader range, from 20.82 ± 0.81 to 143.84 ± 5.56 Bq/m^2^·h, with an average of 49.32 ± 1.91 Bq/m^2^·h. Some areas where local construction activities occur, especially near the shores where sediments are actively used for building, display high ^222^Rn exhalation rates. This highlights their potential contribution to elevated ^222^Rn levels in the immediate environment, increasing exposure to the population. A strong positive correlation (+ 1), as shown in Fig. 5, was observed between the equilibrium Rn_*eq*_ and both ExM and ExA. This indicates that samples with higher ^222^Rn concentrations tend to release ^222^Rn more effectively, both when normalized by mass and area. This correlation reinforces the consistency of measurement methods and the intrinsic relationship between ^222^Rn concentration and its exhalation dynamics. The study of ^222^Rn exhalation rates is critical because it links static ^222^Rn concentration with the dynamic release process. In the study area, characterized by geological formations affecting ^222^Rn activity, these findings indicate that the use of local sediments in construction activities may lead to moderate increases in ^222^Rn exposure, particularly in confined spaces such as small homes or huts built near the lake. For example, sediments used in the construction of homes and fishing huts near the lakeshore may increase exposure to ^222^Rn, particularly in confined spaces such as small homes or huts built in proximity to the lake. Exhalation rates also serve as predictive tools for estimating ^222^Rn dispersion into the atmosphere or confined spaces, contributing to a comprehensive risk assessment framework. By focusing on ^222^Rn exhalation rates, this section complements the previous analysis of Rn_*eq*_ concentrations and Co_*Ra*_, offering a dynamic perspective on ^222^Rn behavior in the area. These results underscore the need for targeted management strategies in the study area, particularly in regions where sediment used for construction is common, to address the environmental and health risks associated with ^222^Rn exposure.

### Emanation coefficient and its implications in ^222^Rn release from the sediments of Nasser Lake

**Fig. 6:**
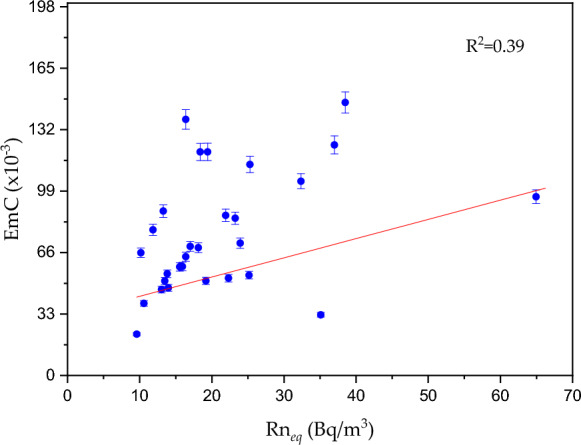
Emanation coefficient versus and equilibrium ^222^Rn concentration for the sediments of Nasser Lake

EmC is a crucial parameter that reflects how efficiently a material releases ^222^Rn due to the decay of ^226^Ra within the sample. This coefficient was determined through the relationship between the chamber volume used for emission measurement and the ^226^Ra activity concentration in the samples. The EmC plays a vital role in environmental research, particularly in evaluating the potential exhalation of ^222^Rn from materials like soil, rock, or construction materials. It is integral to understand the environmental and health implications of naturally occurring radioactive materials in areas such as Nasser Lake, where natural geological factors contribute significantly to ^222^Rn dynamics. The EmC values (× 10^–3^) for sediment samples from Nasser Lake ranged from 22.16 ± 0.86 to 214.98 ± 8.32, with an average of 80.6 ± 3.12. This variability reflects differences in the physical, radiological, and geological characteristics of the sediments, as well as the local environmental conditions. These findings indicate that some areas of Nasser Lake contribute more to ^222^Rn release, likely due to the specific nature of the surrounding geological formations. Figure 6 illustrates the behavior of EmC with the variation of Rn_*eq*_. The plot revealed a weak positive correlation, suggesting that sediments with higher concentrations of ^222^Rn tend to release it at higher rates. However, there were scattered points around the trendline, indicating that the relationship between these two parameters is not perfectly linear. This variability may be attributed to differences in the internal composition of the samples or the unique geological features that influence ^222^Rn exhalation. Understanding the EmC in relation to ^222^Rn concentration is important for assessing its release behavior and potential contribution to indoor and outdoor ^222^Rn exposure, particularly in areas where sediments are used for construction purposes. This knowledge will help in evaluating health risks, especially for local populations living in proximity to areas where these sediments are used in construction or where there is regular human activity.

### Health risk analysis for local residents and fishermen

Calculating the AED from ^222^Rn exposure in open areas is crucial for assessing potential health risks associated with this radioactive gas. While ^222^Rn primarily accumulates in enclosed spaces, its presence in outdoor environments can contribute to radiation exposure, particularly in regions where local communities use sediments rich in naturally occurring radioactive materials for construction purposes. In this study, ^222^Rn concentrations were measured in sediment samples taken from Nasser Lake, a region characterized by active fishing and seasonal rebuilding of homes and huts using lake sediments. The AED was calculated based on experimentally determined equilibrium ^222^Rn concentrations, following the methodology provided by UNSCEAR, which accounts for inhalation exposure under typical residential conditions (United Nations Scientific Committee on the Effects of Atomic Radiation (UNSCEAR), 2000). The AED values ranged from 0.24 to 1.67 mSv/year, with an average of 0.57 mSv/year, reflecting realistic exposure scenarios for local residents and fishermen who are frequently exposed to sediments with varying ^222^Rn levels. These results fall mostly within the internationally recommended safety threshold of ≤ 1 mSv/year for public exposure as defined by WHO (2009) and ICRP (1993) guidelines; however, certain sampling locations near the High Dam showed slightly elevated doses, exceeding the reference level due to higher radium content and emanation characteristics of the sediments. This suggests that while most areas pose minimal radiological risk, specific zones require closer attention and ongoing monitoring. The WHO highlights that ^222^Rn and its decay products are among the leading causes of lung cancer globally, contributing to between 3 and 14% of cases depending on regional exposure levels and lifestyle factors such as smoking (Goher et al., 2021). Although these measurements were conducted in open-air conditions rather than confined indoor environments, the observed variations in ^222^Rn exhalation rates suggest that localized accumulation may occur in semi-confined settings like small fishing huts or homes built with high-emission sediments, thereby increasing the potential exposure to occupants. Given these findings, preventive measures are recommended, especially in areas with elevated AED estimates, including improving ventilation in dwellings constructed with local sediments and raising awareness among residents and fishermen about potential radiation exposure. It is also essential to implement regular monitoring programs to ensure environmental and public health safety. These results emphasize the importance of integrating radiological assessments into local environmental policies, particularly in areas where human activities intersect with naturally occurring radioactive materials. This work contributes to ongoing efforts to develop informed risk mitigation strategies and supports the establishment of appropriate guidelines for managing ^222^Rn exposure in sediment-rich environments like Lake Nasser.

The study underscores the critical role of ^222^Rn and its related parameters—Co_*Ra*_, ExM, ExA, and EmC—in assessing environmental health risks. The results emphasize the significance of understanding ^222^Rn dynamics in sediment-rich regions, such as Nasser Lake, where natural geological factors, like the distribution of radium-rich sediments, have a substantial impact on ^222^Rn concentrations. These parameters provide a comprehensive framework for evaluating the behavior of ^222^Rn and its potential impact on both the environment and human health. Co_Ra_ serves as the primary source of ^222^Rn and plays a central role in predicting its concentration in air, while ExM, ExA, and EmC offer insights into the material’s ability to release ^222^Rn into the atmosphere, affecting its dispersion in local areas, particularly near settlements or fishing activities. The findings are essential for developing effective monitoring strategies and risk mitigation measures in locations that experience minimally direct human intervention but contain considerable levels of naturally occurring radioactive materials. They also highlight the need for continued attention to ^222^Rn levels in areas characterized by active fishing and construction using lake sediments, as these activities may enhance localized exposure under certain conditions. Moreover, the study emphasizes the necessity of continuous environmental monitoring in regions where human practices, such as building homes or huts from sediment materials, could potentially increase radiation exposure. This research contributes to ongoing efforts aimed at establishing science-based guidelines for managing ^222^Rn in natural environments, aligning with global public health protection initiatives. It provides valuable data that can assist environmental and health agencies in formulating evidence-informed policies and practices to minimize ^222^Rn exposure in affected regions. Additionally, it opens the door for future investigations, particularly into the long-term health effects of ^222^Rn exposure in areas with similar geological features, such as those with radium-rich soils and rocks.

## Conclusion

Lake Nasser represents a sedimentary environment rich in minerals and naturally occurring radioactive materials, where radon gas may accumulate due to the presence of radium in sediments. The results reveal considerable spatial variability in radon concentrations across the study area, with maximum and minimum values reaching 66.24 ± 2.56 Bq/m^3^ and 9.59 ± 0.37 Bq/m^3^, respectively, all within internationally accepted safety limits. However, certain locations near the High Dam show relatively elevated levels, indicating the need for continuous monitoring in these zones. A clear positive correlation was observed between radium content and radon concentration, confirming that radon originates primarily from the decay of radium in sediment samples. Radon exhalation rates also varied significantly, reflecting the influence of sediment composition on radon release into the surrounding environment. These findings suggest that areas with higher exhalation potential may contribute more to local radon exposure, particularly when such sediments are used in construction activities. The annual effective dose, calculated using an internationally recognized methodology, ranged from 0.24 to 1.67 mSv/year, with an average of 0.57 mSv/year, mostly falling within the global safety threshold recommended for public exposure. Although these values do not indicate immediate health risks, they highlight the importance of assessing long-term exposure scenarios, especially in semi-confined environments such as small homes or fishing huts built by local communities using lake sediments. This study presents the first comprehensive assessment of radon behavior in Nasser Lake sediments, employing accurate experimental methods to measure radon exhalation and emanation characteristics. It underscores the necessity of integrating radiological risk assessments into local environmental policies, particularly in areas where human activity intersects with naturally occurring radioactive materials. While current levels do not suggest significant hazards, prolonged use of high-emission sediments in residential structures may increase exposure under poor ventilation conditions. Future research should include direct indoor measurements in dwellings constructed from these sediments, as well as investigations into the effects of seasonal variations and moisture content on radon exhalation. Long-term epidemiological studies on local populations are also recommended to better understand potential health impacts. Collectively, these efforts will support the development of science-based guidelines for managing natural radioactivity in sediment-rich ecosystems, contributing to broader initiatives aimed at protecting both human health and environmental integrity.

## Data Availability

No datasets were generated or analysed during the current study.
